# A Scoping Review on COVID-19 Vaccine Hesitancy among the Lesbian, Gay, Bisexual, Transgender, Queer, Intersex and Asexual (LGBTQIA+) Community and Factors Fostering Its Refusal

**DOI:** 10.3390/healthcare11020245

**Published:** 2023-01-13

**Authors:** Jyotsna Needamangalam Balaji, Sreenidhi Prakash, Ashish Joshi, Krishna Mohan Surapaneni

**Affiliations:** 1 Panimalar Medical College Hospital & Research Institute, Varadharajapuram, Chennai 600-123, Tamil Nadu, India; 2 School of Public Health, The University of Memphis, Memphis, TN 38152, USA; 3 SMAART Population Health Informatics Intervention Center, Foundation of Healthcare Technologies Society, Panimalar Medical College Hospital & Research Institute, Varadharajapuram, Chennai 600-123, Tamil Nadu, India; 4 Departments of Biochemistry, Medical Education, Molecular Virology, Research, Clinical Skills & Simulation, Panimalar Medical College Hospital & Research Institute, Varadharajapuram, Chennai 600-123, Tamil Nadu, India

**Keywords:** LGBTQIA+, COVID-19, vaccines, hesitancy, refusal

## Abstract

Background: The ramifications of the existing crisis caused by the coronavirus pandemic are sensed in all walks of life. Among the various efforts made to curb the spread of this novel infection, the development of COVID-19 vaccines had a profound role in flattening the pandemic curve. Even though the rapid vaccine drive received a highly welcoming response among people, the reluctance and ignorance of a part of the population towards available safe vaccines stand as impediments to achieving the desired outcome. The LGBTQIA+ (Lesbian, Gay, Bisexual, Transgender, Queer, Intersex and Asexual) communities are the least studied groups in this regard. Objective: The purpose of this study is to extensively review and report on COVID-19 vaccine uptake and refusal among the LGBTQIA+ population and enumerate the factors contributing to vaccine hesitancy. The study extends further to outline a conceptual framework for interventions to enhance COVID-19 vaccine acceptance among the LGBTQIA+ population. Methods: We performed a systematic search using key terms on Google Scholar and PubMed. The obtained results were filtered using the eligibility criteria framed for this study. The initial search provided an extensive result of 4510 articles which were later screened at various levels to arrive at the final inclusive collection of manuscripts adding to 17. The studies were analyzed by the authors individually, and the data were categorized using variables. The results are interpreted using charts and graphs. The whole manuscript has been structured in accordance with the PRISMA extension for scoping reviews. Result: The comprehensive search yielded 17 eligible articles for this review. Most of the studies were conducted in the United States (n = 17), and predominantly cross-sectional studies have been conducted. The major comparative factor was the HIV status of the LGBTQIA+ population. HIV-affected patients were more willing to take up COVID-19 vaccination. However, social stigma, discrimination, lack of access and non-prioritization in vaccine drives were found to be the major factors contributing to vaccine hesitancy among this population. Conclusion: The invention of the COVID-19 vaccination revolutionized the healthcare systems burdened with COVID-19. Although this is a breakthrough scientific contribution, many factors are associated with the rate of vaccine acceptance, especially among sexual and gender minorities. The reviewed studies have revealed numerous factors that influence vaccine uptake and refusal with the commonest being concerns on discrimination, social stigma, inequitable access to healthcare, vaccine safety, efficacy, potency, side effects and lack of trust in medical workers. These impediments in vaccine coverage should be meticulously addressed to ensure optimum LGBTQIA+ physical and mental health as well as for providing non-discriminative, equitable and quality healthcare service regardless of the gender or sexual orientation of individuals.

## 1. Introduction

The Wuhan Municipal Health Commission reported a cluster of cases with pneumonia-like symptoms in December 2019 [1]. In late January 2020, new cases were detected outside China, in Thailand, Korea and Japan [2]. The World Health Organization designated this novel infection of unknown etiology as the Coronavirus disease 2019 (COVID-19). The global situation was meticulously analyzed, and multitudinous assessments were conducted by the WHO before the director general proclaimed COVID-19 to have the attributes of a pandemic [1,2]. The causative organism of COVID-19 was identified as the severe acute respiratory syndrome corona virus-2 (SARS-CoV-2) virus [1].

Health workers around the world were committed to developing vaccines that were antagonistic towards the SARS-CoV-2 virus. The practicability of vaccines to restrain the spread of COVID-19 infection is not solely subject to vaccine efficacy and protection. Vaccine acceptance and refusal among the global population seem to have a critical role in the successful control of the disease [3]. The SAGE Working Group on Vaccine Hesitancy deduced that “vaccine hesitancy refers to a delay in acceptance or refusal of vaccination despite the availability of vaccination services” [4]. The readiness for vaccination is enormously controlled by concerns over the adverse effects, efficacy and potency of the vaccine. Widespread mistrust of healthcare providers and the healthcare system reinforced vaccine refusal. Ethnicity, employment status, personal beliefs, religion, politics, gender, education, age, income, prior exposure to COVID-19 and accessibility to healthcare facilities are some of the key elements determining the rate of vaccine acceptance [5].

Substantial progress has been made in investigating diverse factors contributing to vaccine acceptance and refusal to a great degree via survey-based study models. Nonetheless, it is critical to note that the marginalized communities of our society—the lesbian, gay, bisexual, transgender, queer, intersex and asexual populations who are collectively addressed under the inclusive term LGBTQIA+ owing to their gender and sexuality—are left unnoticed in these studies, or that due importance is not given to this population subset in vaccine hesitancy studies targeting the general population [6]. The COVID-19 pandemic has inordinately affected sexual minorities across the globe. The LGBTQIA+ population is one of the groups more susceptible to HIV infection and other co-morbid conditions. This made them vulnerable to severe COVID-19 infection. Additionally, the impacts of socioeconomic downfall, mental stress, inequitable access to healthcare and discrimination are prime factors that hold back this marginalized community from becoming vaccinated [6].

In spite of the development of a safe and highly effective COVID-19 vaccine, ample hurdles to vaccine acceptance and deployment obstruct the efficacy of vaccines in controlling the spread of this novel disease. Particularly, the marginalization of LGBTQIA+ communities renders this population disproportionately vulnerable to COVID-19 morbidity, mortality and unlikeliness to become vaccinated [7]. Several factors are known to contribute to vaccine hesitancy among the LGBTQIA+ population. One main issue is the COVID-related anxiety and behavioral changes that invariably impact vaccine acceptance. The prevalence of stress, the fear of side effects, uncertainty in vaccine safety and pandemic suffering collectively affect the mental health of people and are known to have a greater effect on the marginalized and stigmatized population. These factors are inversely associated with the rate of vaccine uptake and are known to be the major cause of vaccine hesitancy among the LGBTQIA+ population [8]. More than 50% of young people in sexual and gender minorities in the USA have reported worsening anxiety or depression symptoms since the COVID-19 pandemic began. The absence of family support, isolation from support networks and disruptions to health care are factors that are likely to be involved in such results [9,10].

Although several studies have identified the factors responsible for vaccine hesitancy among the LGBTQIA+ population, the categorization of factors under separate domains to understand the cause and interventions to address the same have not been substantiated. The clear stratification of factors at various levels is necessary to design and implement appropriate strategies for creating an inclusive environment for the LGBTQIA+ population and achieving universal vaccination. To this rationale, this scoping review was conducted to understand the various determinants and factors influencing vaccine hesitancy in this population, providing crucial information to the healthcare professionals, service providers and policymakers that can help them extend vital support to the LGBTQIA+ communities. This study fills the gap in knowledge by categorizing various factors fostering vaccine refusal and provides an extensive summary of vaccine hesitancy among the LGBTQIA+ population and the underlying factors contributing to it along with an explicit conceptual framework to overcome vaccine hesitancy among the LGBTQIA+ population.

### Objectives of the Study

This scoping review seeks to (i) conduct a comprehensive literature search on COVID-19 vaccine hesitancy among the LGBTQIA+ community, (ii) report on the factors that hinder the vaccine acceptance rate, (iii) elaborate on the need to take necessary steps to enhance the rate of vaccination among the LGBTQIA+ individuals and (iv) provide a conceptual framework with possible suggestions for implementing interventions that will improve the vaccine uptake among this population and promote an inclusive health care environment.

## 2. Materials and Methods

The content of this scoping review embraces the information extracted from manuscripts describing the factors affecting COVID-19 vaccine uptake among the LGBTQIA+ communities from profound databases such as Google Scholar and PubMed. The configuration of this article follows the PRISMA extension for scoping review (PRISMA ScR) [11].

### 2.1. Stage 1: Source of Information

An explicit search was performed in databases Google Scholar and PubMed to identify relevant sources of information. Scientific publications in the English language were selected.

### 2.2. Stage 2: Search Strategy

Pertinent articles from the year 2021 were selected. Key search terms such as ‘COVID-19′; ‘COVID-19 vaccine hesitancy’; ‘LGBTQIA+ communities’; ‘vaccine uptake barriers’; ‘lesbians’; ‘gay’; ‘bisexuals’; ‘transgender’; ‘queer’; ‘vaccine rollout’; ‘inequalities in vaccine drive’; and ‘sexual and minority groups’ were used to filter the results. 

### 2.3. Stage 3: Process of Selection

**Figure 1:**
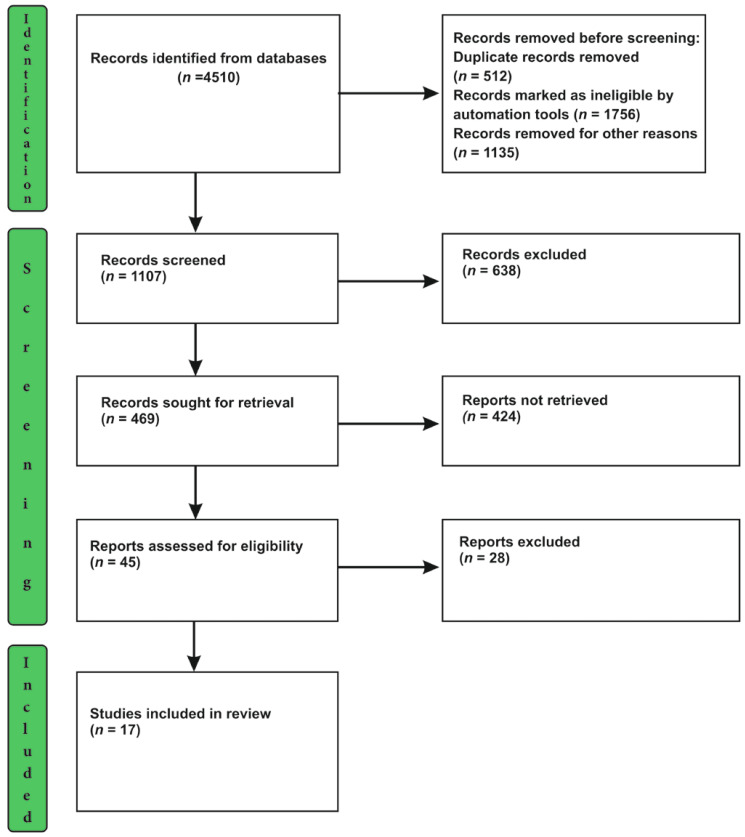
PRISMA Flow Chart.

The process of slection includes three distinct steps, viz. Identification, screening and inclusion of studies. The process of selection has been detailed and depicted in the Figure 1.

### 2.4. Eligibility Criteria

Articles to be encompassed in this scoping view were sorted based on the following inclusion/exclusion criteria. 

#### 2.4.1. Inclusion Criteria

- Scholarly articles published from the year 2020 onwards;
- Scientific literature discussing vaccine hesitancy among the LGBTQIA+ population only
- Studies investigating hesitancy to be vaccinated against coronavirus only were considered for this study;
- Quantitative, qualitative and mixed methods studies were encompassed for analysis;
- Studies conducted in high-, low- and middle-income countries were encompassed for this study;
- Research articles published in peer-reviewed, indexed journals and abstracts;
- Studies correlating the HIV status of the target population and vaccine hesitancy;
- Manuscripts with a core theme of COVID-19 vaccine hesitancy and barriers to acceptance;
- Studies published in only the English language.

#### 2.4.2. Exclusion Criteria

- Articles published before 2021;
- Studies concerned with vaccine hesitancy other than COVID-19 vaccines;
- Scientific literature discussing vaccine hesitancy among the general population or populations other than the LGBTQIA+ community;
- Manuscripts published in languages other than English.

### 2.5. Data Charting

An elaborate data chart comprising all-inclusive variables was prepared and perused by the authors independently. A thorough review was performed analyzing the reliability of the data entered and was charted as per the objectives of this scoping review. 

### 2.6. Data Items

An in-depth analysis of the selected manuscripts was performed, and the data to be extracted were cataloged into name of author, country of study, year of study, aim, study design, sample size, comparison, name of vaccine, HIV status and result.

## 3. Result

### 3.1. Selection of Source of Evidence

From the 4510 results of the search using key terms, the duplicate and ineligible records were removed from consideration to further the process of selection. With 1107 articles, screening was performed by the authors independently to sort out relevant publications for this study. The process of the selection of the source of evidence is explained in Table 1

### 3.2. Characteristics and Results of Source of Evidence 

The table below represents the assemblage of relevant variables which were analyzed for this review. (Table 2).

### 3.3. Summary of Charted Data

#### 3.3.1. Characteristics of Charted Data

As a result of the intricate methods of screening, 17 articles were selected for this study. Our analysis puts forth the fact that COVID-19 vaccine acceptance is still suboptimal among the LGBTQIA+ communities. The studies included in this review were predominantly conducted in the United States (n = 11) with the least number being conducted in Europe (n = 1). We included different types of study design such as (i) Cross-sectional study (n = 10); (ii) prospective cohort study (n = 3); (iii) mixed methods study (n = 2); (iv) qualitative study (n = 1) and (v) systematic review (n = 1). It is also to be noted that most of the studies included in this review have been published recently in 2022 (n = 9), followed by 2021 (n = 7) and one study in 2020 (n = 1). Considering the HIV status of the individuals included in the studies, most of the studies have not mentioned the HIV status of their study population (n = 9), few have included both HIV-positive and negative participants (n = 5), while two studies considered only HIV-positive respondents (n = 3). 

**Figure 2:**
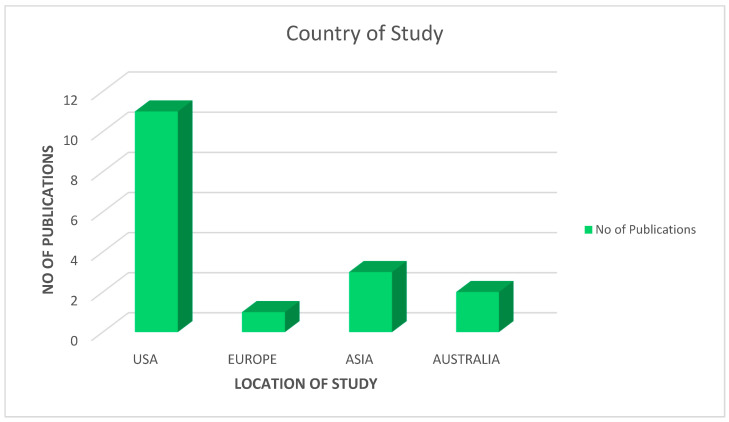
No. of publications from different countries.

Figure 2 represents the number of publications that have been included in this review from different geographical regions.

**Figure 3:**
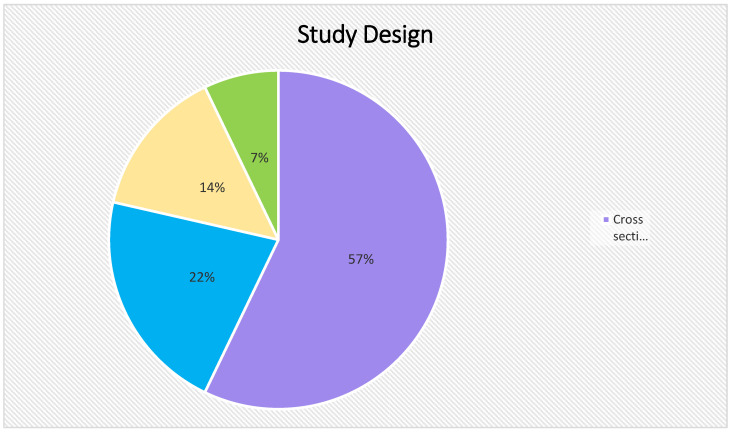
Different types of study designs.

Figure 3 shows the different types of study designs included in this scoping study. 

**Figure 4:**
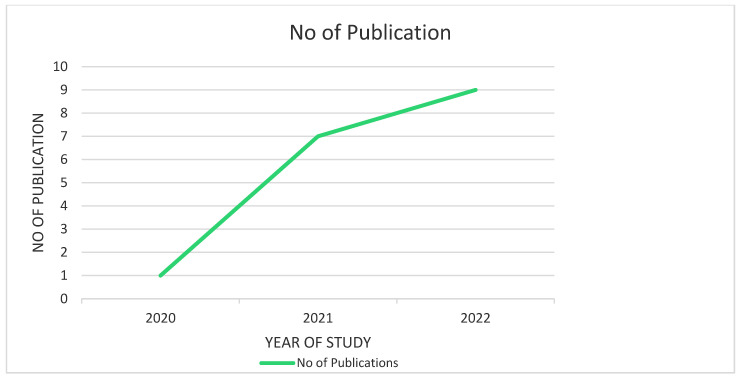
No. of publications in the last 2 years included in this study.

Figure 4 portrays the number of scientific studies published in the last two years which have been analyzed in this review. 

**Figure 5:**
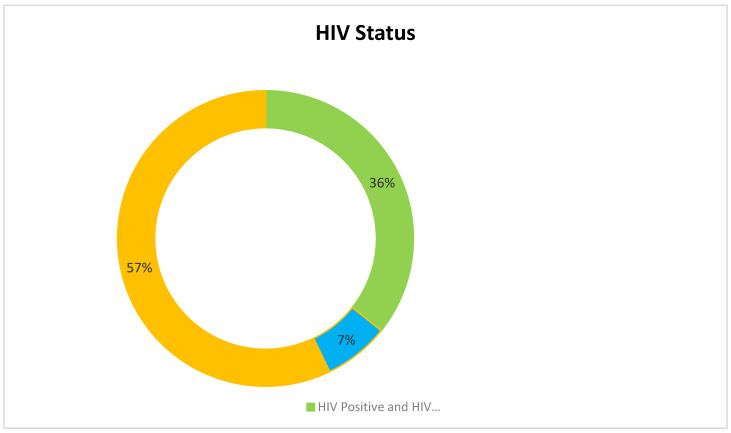
HIV status of study participants.

Figure 5 elaborates on the HIV status of the study participants of the studies taken into consideration for this review. 

**Figure 6:**
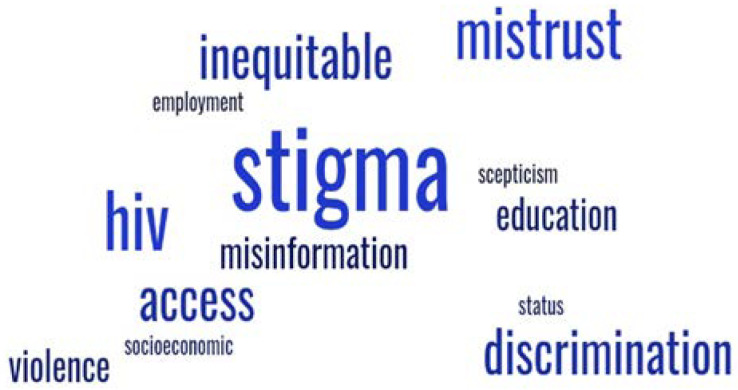
Keywords used in the articles selected for this review.

Figure 6 depicts the summary of keywords used in the articles selected for this review

#### 3.3.2. Results of Factors Influencing Vaccine Uptake among LGBTQIA+ Population

Several factors contribute to the vaccine hesitancy among the LGBTQIA+ community (Table 3). They can be categorized into individual, interpersonal, social and community, healthcare system-related and vaccine-related factors. Age, ethnicity, education, geographical location, occupation, income level, marital status, HIV status, knowledge and awareness, perceived risks, beliefs and attitudes are found to be the individual factors that affect the vaccine uptake. Additionally, the social stigma and discrimination faced by them which causes social isolation and a lack of support and guidance may increase the likelihood of vaccine negligence. Their exclusion from the general population causes unemployment and poverty and makes the healthcare system less specific towards LGBTQIA+ health leading to rejection and discrimination. These inequalities in health care and past unacceptable and depressing experiences with healthcare services cause medical mistrust among this population. In addition to these, factors such as new vaccine introduction, the risk associated with it, the design of the vaccine program, the reliability of the vaccine, cost, safety, efficacy and availability were found to affect the rate of vaccine uptake. Additionally, the non-allocation of vaccines to sexual and gender minorities was found to be one of the major factors that contributed to reduced vaccine uptake among the LGBTQIA+ community. The non-allocation of vaccines, the exclusion of this population from the vaccine schedule and the lack of proper transfer of information were found to be associated with higher vaccine hesitancy. 

## 4. Discussion

### 4.1. The Current Scenario

Abstaining a person from health benefits that need to be offered because of his/her sexual orientation is certainly unfair. However, stigma and discrimination towards sexual and gender minorities have led to health disparities [29]. A massive step taken towards the control of the COVID-19 pandemic is the vaccination drive. The chief objective of this is to make sure every eligible citizen is vaccinated and in safe conditions. However, the lack of accessibility and acceptance makes a group of the population vulnerable to health hazards. The major factor attributed to vaccine hesitancy among the LGBTQIA+ communities is the medical mistrust, trauma and social stigma [12,21]. Evidence also suggests that the socioeconomic factors, educational qualifications and employment status of individuals in this community play a key role in determining the willingness towards getting vaccinated [13,14,17]. The health status of sexual and gender minorities also acts as a precipitating factor in providing motivation towards vaccine uptake. As the LGBTQIA+ population is at a greater risk of getting infected, HIV-positive individuals show an optimistic response to becoming vaccinated [14,15]. Reducing the burden of skepticism and altruistic attitudes is also known to influence the likelihood of being vaccinated [16,18,21]. Nevertheless, the fear of side effects, vaccine insufficiency and the safety of newly developed vaccines is also a cause for concern in this regard [19]. On a positive note, it is worth noting that sexual and gender minorities are intrinsically willing to receive their jabs and show increased interest towards enhancing their health literacy [20]. Survey-based studies conducted in the US have revealed that 85% of the gay and lesbian population have received at least a single dose of the COVID-19 vaccine against 76% of heterosexual individuals [30]. However, the overall vaccination coverage among this population on a global scale is still found to be suboptimal compared to the general population [23,24]. The LGBTQIA+ population frequently delineates obstacles in accessing good quality healthcare [31,32]. A detailed investigation of this hurdle plays a crucial role in achieving sustainable development goals which advocate gender equality and good health and well-being [33]. The emergence of COVID-19 has prepended yet another pressure that has adversely impacted this group of sexual minority populations [34]. As immunization is the most virtuous and affordable health intervention to curb the spread of communicable diseases, COVID-19 vaccination plays an eminent role in controlling the transmission of this novel infection [23]. Owing to the widespread existence of co-morbidities, individuals belonging to the LGBTQIA+ communities are relatively at a greater risk of acquiring COVID-19 infection predisposing them to a dire need for vaccination [35].

### 4.2. Common Elements Influencing Vaccine Acceptance and Refusal

The main challenge faced in the path of achieving global vaccination coverage is vaccine hesitancy. Unbiased access and the unanimous acceptance of safe and effective vaccines are the key strategies to bringing the COVID-19 pandemic to an end [20]. Diverse factors are known to influence the rate of vaccine acceptance and refusal. Similar to the concerns of the general population regarding vaccine safety, the fear of adverse effects, efficacy and potency, these factors also influence the vaccine’s acceptance rate among the LGBTQIA+ population [15,21]. Other personal factors that influence vaccine uptake which are specific for individuals that appear to be common between the LGBTQIA+ and general populations are stress, depression, anxiety, unemployment, homelessness, educational qualification and socioeconomic status [25,36,37,38]. 

### 4.3. Determinants of Vaccine Hesitancy Specific to the LGBTQIA+ Community

The determinants which affect the willingness towards being vaccinated that are peculiar to the LGBTQIA+ communities are systemic discrimination, stigma, social isolation, medical mistrust resulting in inequitable access and reluctance to health care and the HIV crisis [39,40,41,42,43,44].

#### 4.3.1. Systemic Discrimination

Discrimination in healthcare facilities harms LGBTQIA+ individuals’ lives via denials and delays of medical assistance. Despite various laws and legal frameworks to protect this vulnerable population, individuals of the LGBTQIA+ community encounter healthcare discrimination ranging from harassment and ignominy to denial of healthcare services [45]. In a society where heterosexuality is the default and the norm, LGBTQIA+ individuals face rejection by healthcare providers on the grounds of their perceived sexual orientation [46]. This stands as a barrier to LGBTQIA+ individuals in accessing healthcare services and a potential deterrent to COVID-19 vaccine acceptance among this population.

#### 4.3.2. Social Stigma

Stigma can be interpreted in three main domains—enacted stigma, anticipated stigma and internalized stigma. Each of these has its own influence on health-related behaviors. Enacted stigma is due to the absurd external attitudes and unfair behavior of others towards an individual. This causes anticipated stigma among people, which makes them believe that they are the targets of prejudice and discrimination in society which results in a reluctance to access healthcare services. Internalized stigma changes the way one thinks about his/her own self, capable of lowering self-esteem, and may lead to negative health behaviors such as tobacco and substance use leading to worse mental health outcomes and hesitancy to approach medical facilities for healthcare assistance. Social stigma is thus a crucial factor to be considered in vaccine acceptance and refusal [47].

#### 4.3.3. Medical Mistrust

The above-discussed stigma and discrimination towards the LGBTQIA+ community are also evident among the medical profession. Clinicians and other healthcare professionals are lacking in cultural competencies in offering humanistic care to LGBTQIA+ patients due to the non-inclusion of LGBTQIA+-specific competencies in the medical curriculum and inadequate exposure to effective communication and empathetic service to stigmatized populations. This ambiguity and uncertainty in the way healthcare professionals treat them with preconceived stigma create a sense of insecurity among LGBTQIA+ individuals that their health needs are not understood by their doctors and that they are not able to address their health needs, thereby heightening the levels of medical mistrust among the LGBTQIA+ population [48]. Thus, LGBTQIA+ patients have a sense of distrust of the medical system overall, which results in poor rates of vaccine acceptance. While several research studies have investigated the impact of mistrust in medical services, there is a paucity of studies correlating this with COVID-19 vaccine hesitancy among this population [49].

#### 4.3.4. HIV Status

Although the LGBTQIA+ population has substantial reasons for their mistrust and negligence to approach healthcare providers due to intolerant attitudes and demoralizing experiences, they are also more likely to show optimistic adherence to health advice, public health interventions and treatment protocols. This is particularly evident during any disease outbreaks owing to their higher vulnerability to acquiring diseases, and they show frustration when they are not availed of the public health benefits that they deserve [50]. In support of this, vaccine acceptance as a prophylactic strategy to protect themselves and their sexual partners from infection is reported to be higher among the individuals of the LGBTQIA+ community [20]. LGBTQIA+ patients who are identified to be HIV-positive have a higher rate of COVID-19 vaccine acceptance and are more motivated to become vaccinated in the initial phases of vaccine rollout as compared to individuals who are not tested for HIV or are unaware of their HIV status or HIV-negative individuals [14,15,38]. 

### 4.4. The Need of the Hour

Comprehensive and meticulously planned health interventions are the need of the moment to foster the health of the LGBTQIA+ population. Building up trust, enhancing health literacy, improving data collection, properly reporting data, unbiased approach, the inclusion of competencies regarding offering equal health service to LGBTQIA+ communities and uprooting social stigma towards this population may significantly contribute towards vaccine acceptance [35]. These can be achieved by enhancing cultural responsiveness training, providing deep insights about sexual orientation and gender identity measures, conducting studies to figure out the impact of COVID-19 on the LGBTQIA+ population and including them in disaster management programs without bias [51]. 

### 4.5. Mental Health and Vaccine Hesitancy

During the COVID-19 pandemic, mental health issues are common, such as perceived stress and sadness, which may have a detrimental influence on perceptions about COVID-19 vaccination or vaccination intention. Poor mental health may cause people to have more negative attitudes regarding the vaccine and be less inclined to receive it. This mental disturbance was found to have the most influential role in vaccine hesitancy. The LGBTQIA+ population is no exception to this [52]. This builds the exigent need to have more mental health providers to support and care for the LGBTQIA+ population during the pandemic. In order to assist patients in overcoming obstacles, mental health practitioners and teams are educated to use empathy, reflective listening and cooperative goal planning. These experts aggressively promote patients’ health and encourage them to adopt healthy habits including being vaccinated against COVID-19 [53].

### 4.6. LGBTQIA+ Inclusivity in Vaccination and Public Health 

The universal vaccination drive with LGBTQIA+ inclusion undoubtedly contributes to the enhancement of public health. Efforts should be taken to improve awareness about personal and public health. People can better comprehend the effects of health inequities in the LGBTQ community by being informed, educated and empowered. A clear conceptualization of the factors contributing to vaccine hesitancy is highly essential for examining the underlying causes of poor health outcomes, creating connections with the community and connecting the LGBTQIA+ population with services and resources in order to end health inequities in the LGBTQ community and designing policies to encouraging community involvement [54]. Working towards this goal requires public health professionals to include people of all sexual orientations and gender identities. Public health professionals play a critical role in correcting the worrisome healthcare disparities encountered by the LGBTQIA+ community through teaching, engagement, and cooperation within organizations, communities, institutions and other public health agencies and healthcare professionals [55]. This study thus provides a comprehensive review of the factors that foster vaccine hesitancy among the LGBTQIA+ population and also conceptualizes a framework in order to design and develop interventions that can be used by policymakers, stakeholders and other health professionals in order to improve vaccine uptake and create a non-discriminative health care system

## 5. Conclusions

This scoping review concentrated on COVID-19 vaccine hesitancy among the LGBTQIA+ population in different countries across the globe. The study focused on establishing the factors that make up for this refusal. The LGBTQIA+ population lives in a biased, stereotyped and prejudiced environment. They are subjected to discrimination, oppression, mental stress and, more importantly, health inequities. Their unheard grievances heightened further during the pandemic. As this population was not given due consideration in vaccine studies, there is a paucity of authentic data on disproportionate access to healthcare facilities and the COVID-19 vaccine. Upon systematically analyzing the available data, it was discovered that concerns about the safety, side effects and efficacy of vaccines, mistrust in healthcare providers, discrimination based on gender identity, a lack of targeted information about vaccines and the inequitable allocation of the vaccine stood as potential barriers to vaccine uptake in this community. Moreover, individuals belonging to LGBTQIA+ communities with low levels of education or being socioeconomically backward or unemployed had a lower rate of vaccine acceptance. No notable difference was found in the rates of vaccine acceptance between the LGBTQIA+ community and the general population. However, a noteworthy disparity is seen in the availability and access to the COVID-19 vaccine in the LGBTQIA+ population. With new variants emerging, it becomes inevitable to ensure that this population is not left behind but is well immunized. Our study provides deep insights about the various determinants of vaccine acceptance and refusal among the targeted population, which will lend a helping hand to health care providers and policymakers in planning appropriate vaccination schemes for the LGBTQIA+ population.

### 5.1. Knowledge Gaps

Even though numerous studies have been conducted analyzing the COVID-19 vaccine hesitancy among the general population, there is an acute paucity of data that unveils vaccine acceptance and refusal among the marginalized LGBTQIA+ population. Certain proposed factors such as social stigma, discrimination and HIV status have not been investigated individually. Certain determinants that impact vaccine uptake are common to the LGBTQIA+ community and the general population such as socioeconomic status, age, educational status, etc. Despite this similarity in factors, significant differences exist in influencing the vaccine acceptance in the population which remains unexplored. Additionally, evidence-based health interventions that could foster vaccine uptake in the LGBTQIA+ population are left undiscovered. The health disparities faced by LGBTQIA+ and the inequitable allocation of resources and vaccines, especially during the COVID-19 pandemic, are not comprehensively addressed in most of the studies. Moreover, studies conducted so far do not provide substantial information about the factors that motivated LGBTQIA+ individuals who have become vaccinated.

### 5.2. Limitations

Although the content of this scoping review embraces the majority of the issues considered as barriers to COVID-19 vaccine uptake among the LGBTQIA+ population, information extracted from articles published only in the English language may stand as a limitation in skipping the results published in other languages. It is also to be noted that published manuscripts from only two databases, Google Scholar and PubMed, were included in this review. 

### 5.3. Directions of Future Research

Vaccine hesitancy is not merely due to the personal perception of an individual but also because of the challenges in registration, vaccination and follow-up faced by them, although they are ready to utilize the available health advancements. Steps should be taken to eradicate the stereotypes faced by LGBTQIA+ individuals and to promote an inclusive environment for them. Additionally, more elaborate research on special measures that must be in place to avail this population of better healthcare services should be conducted. Analyzing not only the barriers but also bringing to light interventions that can benefit this population will enhance their standard of living and abolish fear and humiliation, promoting better health facilities without any bigotry.

## 6. Conceptual Framework

Based on a comprehensive review of existing literature and by critically looking at the issue in the context of the ongoing COVID-19 pandemic, we put forth two strategies to conquer vaccine hesitancy among the LGBTQIA+ population:

I. Inclusive healthcare environment;
II. Digital health interventions.

### 6.1. Rationale for Developing an Inclusive Healthcare Environment and Digital Health Interventions to Address Vaccine Hesitancy among LGBTQIA+ Individuals 

The major cause of the medical mistrust and lack of participatory behavior among the LGBTQIA+ population is the existence of health disparities that arise due to stigmatization and discrimination. Thus, the establishment of an inclusive healthcare environment for the LGBTQIA+ population is the first step towards eradicating any health inequalities in this community. An inclusive healthcare environment along with compassionate, respectful and empathetic treatment will pave the way for a more humanistic medical service that will improve health outcomes and the adherence of patients to health advice and treatment measures. In addition to this, the recent advancements in technology and the COVID-19 pandemic have set the stage for digitalization both in terms of patient care and health administration. Personalized and inclusive care for the LGBTQIA+ population can be promoted by implementing digital health interventions such as the development of mHealth applications for vaccine coverage exclusively for the LGBTQIA+ population. This will facilitate the vaccination drive by providing information about vaccines and the need for vaccination, creating awareness about newly developed vaccines, enhancing access to vaccination through online portals that show the availability of vaccination centers nearby and prior registration, monitoring health and vaccination status, regular reminders for vaccination dues and resolving any queries of the users.

### 6.2. Inclusive Healthcare Environment

An LGBTQIA+-inclusive healthcare environment can be promoted by addressing the key issues such as communication and the fear of discrimination. These elements have been discussed at the healthcare worker level, the organizational level and the community level, which explains the methods of promoting inclusivity and the intended outcomes of these actions (Table 4).

### 6.3. Digital Health Interventions

The proposed framework for digital health interventions aids healthcare professionals and policymakers in appropriately utilizing health technologies for improving and enhancing vaccination programs. It facilitates the active engagement of LGBTQIA+ individuals, thus establishing a personalized and inclusive healthcare delivery system. Communication of authentic and reliable vaccine-related information is of immense importance to prevent misconceptions regarding newly developed vaccines. Health information technologies such as health portals, SMS, etc. are vital tools for transmitting reliable information pertaining to COVID-19 vaccines. This in turn instills confidence about COVID-19 vaccines, thereby improving the attitude of LGBTQIA+ individuals towards vaccination. Additionally, for the successful implementation of this intervention, accurate vaccination records and contact information should be maintained in the electronic health record system.

### 6.4. Development of “mHealth” App for Facilitating Vaccination Programme for LGBTQIA+ Population

Mobile health technologies are widely used for data collection, the maintenance of records, patient communication, treatment, follow-up and reminders. Moreover, evidence has proven the successful implementation of mHealth interventions via a mobile phone application to increase the adherence and follow-up of patients to healthcare facilities via reminder notifications. Hence, we strongly recommend the development of a mobile phone application explicitly for promoting COVID-19 vaccination among the LGBTQIA+ population. We put forth the following recommendations for an “mHealth” app’s development and implementation explained in Table 5.

1. Efficient mobile application development team;
2. Effective designing of the mobile application;
3. Testing of the prototype application;
4. Components and features of the mobile application;
5. Assuring privacy and data security.

### 6.5. Barriers to Promote Inclusivity and Implementation of Digital Health Technologies

The successful implementation of the proposed conceptual framework for reducing vaccine hesitancy among the LGBTQIA+ community can be hindered by potential barriers to promoting inclusivity and digital health technologies. The following table provides an overview of the social, cultural and structural barriers identified through a critical review of the literature (Table 6).

The main drawback in promoting LGBTQIA+ inclusivity is the discrimination and stigma that dominate in the minds of people. Personal beliefs and behaviors tend to affect the rate of acceptance and inclusion of sexual and gender minorities. Thus, clearing this bigotry among the people remains the main area of focus for promoting inclusivity. In addition to this, the workforce must be led by an eminent leader who can work towards LGBTQIA+ health issues and recruit skillful members to form the team, an ideally functioning unit to achieve the goal of LGBTQIA+ inclusion. Thus, administrative support is highly essential for implementing any intervention for public benefit. On the other hand, the challenge in the implementation of digital health technologies lies in the proper designing, developing and delivering of health technologies at affordable prices to the targeted population. Although traditional methods are being rapidly replaced by digital interventions, the lack of awareness, lack of trust and non-adaptable mindset of certain groups of people reduce the overall acceptance and utility of these technological benefits. Additionally, apps and other software developers should consider usability, complexity, language and accessibility as important barriers in developing and successfully delivering digital health technologies, as the goal is to cover all gender minority individuals regardless of their literacy, economic status or residence. Another vital area to consider is the ethical and legal regulations while developing digital technologies. Many ethical considerations such as privacy, security, data piracy fear, reliability and approval from stakeholders, governing bodies and policymakers come into the picture while implementing digital technologies for health benefits. Lastly, the proper implementation of both the inclusivity approach and digital health technologies requires stable financial support, without which, the long-term sustainability of the intervention is highly unlikely, and the system can collapse without funds. Thus, it is highly crucial to consider these barriers and address them effectively to achieve the intended outcomes. 

## Figures and Tables

**Table 1:** Selection of Source of Evidence.

Stage of Screening	Total No of Articles Reviewed	Articles Included	Excluded Articles	Rationale for Exclusion
Title Screening Stage	1107	469	638	Irrelevant to the objective of this study
Abstract Screening Stage	469	45	424	Did not qualify the eligibility criteria set up for this study (given in Section 2.4.1)
Full-Text Screening	45	17	28	Articles did not provide the required information for vaccine hesitancy among LGBTQIA+ and book\book chapters, comments, editorials, letters

**Table 2:** Extracted data.

Sl. No	Reference	Author	Country	Year	Aim	Study Design	Population (Sample Size)	HIV Status of Individuals Involved in the Study	Major Findings
1	[12]	Danny Azucar	USA	2022	To delineate the factors affecting vaccine uptake in the LGBTQIA+ population.	Qualitative study	32 individuals from LGBTQIA+ communities	Not specified	Medical trauma, stigma, discrimination and violence are the major factors contributing to vaccine hesitancy
2	[13]	Andrea Low	USA	2022	To collate COVID-19 vaccine uptake among LGBTQIA+ communities to the general population	Cross-sectional study	Self-identified LGBTQIA+ individuals older than 18 years of age	Not specified	Education, socioeconomic status, medical mistrust, less integration, stigma and discrimination are the strongest determinants of vaccine hesitancy
3	[14]	Martin Holt	Australia	2022	To analyze the level of willingness to be vaccinated	Cross-sectional study	1280 bisexual and gay individuals	HIV-positive or negative	Education, employment and socioeconomic status are established factors affecting vaccine acceptance. HIV-positive individuals show a higher rate of vaccine acceptance
4	[15]	Rob Stephenson	USA	2021	To determine the factors associated with beliefs about the COVID-19 vaccine	Cross-sectional study	Bisexual, gay and men who have sex with men above 18 years of age	HIV-positive or HIV negative	HIV-positive individuals have greater levels of vaccine optimism
5	[16]	Elliott R. Weinstein	USA	2022	To discover the factors related to vaccine likelihood and uptake	Mixed method study	Latino sexual minority men	Not specified	Altruistic motivations were influential in likelihood and vaccine uptake
6	[17]	Youssoufa M. Ousseine	France	2022	To explore the elements causing vaccine uncertainty and unwillingness	Cross-sectional study	Homosexuals, bisexuals, or men who have sex with men aged 18 years or older	HIV-positive or negative	Socioeconomically at-risk individuals are highly reluctant to be vaccinated
7	[18]	G. Prestage	Australia	2022	To recognize factors contributing towards COVID-19 vaccination and contrast sexual behavior pre- and post-vaccination	Prospective, cohort study	Men aged 16 years or above, identified as gay or bisexual or had sex with men	Not specified	Skepticism was a hurdle to vaccine uptake
8	[19]	Alex Abramovich	USA	2022	To enumerate the facilitators and barriers of vaccine uptake among the LGBTQIA+ population	Mixed method study	922 LGBTQIA+ individuals experiencing homelessness	HIV-positive and negative	Mistrust in healthcare, paucity of targeted vaccine-related information, vaccine adverse effects and inequitable access are some of the attributes of vaccine refusal
9	[20]	Yen Ju Lin	Taiwan	2021	To differentiate the levels of explicit and intrinsic intentions to become vaccinated	Prospective cohort study	1047 participants aged 20 years or older	Not specified	Sexual minority populations have greater levels of explicit and intrinsic intentions to accept COVID-19 vaccine uptake
10	[21]	Daniel Teixeira	USA	2021	To assess the vaccine acceptancy among sexual and gender minorities	Cross-sectional study	1350 SGM participants	Not specified	Factors such as medical mistrust, altruism, race, and social concern affected the vaccine acceptancy among this population
11	[22]	Brooke A. Levandowski	USA	2022	To study the nonmedical impact of COVID-19 on LGBTQIA+	Cross-sectional survey	1362 LGBTQ+ participants	Not specified	Public health interventions should be made to countercoup the increased stress among LGBTQIA+ communities to avail them of all resources
12	[23]	Kechun Zhang	China	2022	To analyze the factors that are involved in the vaccine uptake among MSM in China	Prospective cohort study	420 participants	Not specified	The COVID-19 vaccine uptake among this population was identified not to be poor when compared to the general population. Tapping the factors that increase vaccine acceptance will enhance the coverage
13	[24]	Weiran Zheng	China	2021	To assess the barriers to COVID-19 vaccine uptake among HIV-infected MSM in China	Cross-sectional survey	1295 participants	HIV-positive	The vaccine acceptancy among the LGBTQIA+ population is still sub-standard. Addressing the barriers will improve hesitancy.
14	[25]	Gregory Philips	USA	2021	To analyze the impediments in achieving total COVID-19 vaccine coverage among sexual and gender minority groups	Cross-sectional survey	932 participants	HIV-positive and HIV-negative	The factors that stand as challenges need to be addressed via public health measures to improve the standard of living of the SGM population
15	[26]	Ishan Garg	USA	2021	To determine the factors contributing to vaccine hesitancy among the LGBTQIA+ population	Systematic review	Not applicable	Not specified	The LGBTQIA+ community has undergone discrimination, oppression and health inequities which elevated the vaccine hesitancy among this population
16	[27]	Dallas Swendeman	USA	2020	To investigate the attitude of SGM youth towards COVID-19 vaccines	Cross-sectional study	440 individuals	HIV positive	Treatment abuse, incarceration and homelessness contribute to vaccine hesitancy. SGM youth-targeted vaccine campaigns provide promising results in alleviating vaccine hesitancy
17	[28]	Jessica Jaiswal	USA	2021	To Determine the factors contributing to vaccine hesitancy among the SGM population living with HIV	Cross-sectional study	496 HIV-positive individuals	HIV positive	Education, HIV status and higher perceived risk for COVID-19 vulnerability foster vaccine acceptance among the LGBTQIA+ population

**Table 3:** The major factors contributing to vaccine hesitancy among LGBTQIA+ communities.

Individual Factors	Interpersonal Factors	Social and Community Factors	Healthcare System-Related Factors	Vaccine Specific Factors
♦ Age ♦ Ethnicity ♦ Education ♦ Geographical location ♦ Occupation ♦ Income level ♦ Marital status ♦ HIV status ♦ Knowledge and awareness ♦ Perceived risks beliefs and attitude	♦ Social isolation ♦ Lack of support system ♦ Lack of guidance and assistance from family and friends	♦ Social stigma ♦ Discrimination ♦ Unemployment ♦ Poverty ♦ Cultural beliefs ♦ Social norms and values	♦ Paucity of knowledge on LGBTQIA+ specific health requirements ♦ Medical mistrust ♦ Rejection by healthcare professionals ♦ Past experience with healthcare services	♦ Introduction of new vaccine ♦ Risk/benefit ♦ Design of vaccination program ♦ Reliability and source of vaccine supply ♦ Vaccine allocation ♦ Vaccine schedule ♦ Cost

**Table 4:** The major elements identified for promoting inclusivity and intended outcomes of these actions.

Level of Action	Elements	Purpose and Methods	Intended Outcomes
Healthcare Worker Level	Actively engaging Leader	To represent the community that works towards LGBTQIA+. Ensure that the team has sufficient time and access to resources in order to achieve the desired goals and objectives.	Regularly monitored planned activities. Implementation of suggestions based on the evaluation and feedback to build a more welcoming environment for the LGBTQ community to improve vaccine hesitancy.
	Communication and Care	To train the healthcare staff and other workers in healthcare settings for affirming and respectful communication. To impart value-based education to enhance humanization in healthcare systems.	Improved LGBTQIA+ healthcare with greater adherence to the healthcare services. Proper communication related to vaccine information, availability, dosage, advantages, adverse effects and cost.
Organizational Level	Non-discriminative policies forms	Revising the medical history forms and registration forms by extending the gender identity options beyond male and female. Inclusion of spouse or partner option in forms instead of wife or husband and changing parents or guardians instead of mother or father will promote gender inclusivity and eliminates awkwardness and fear of the LGBTQIA+ population from choosing their gender identity.	Improved trust among the individuals of the LGBTQIA+ community to facilitate a feeling of inclusivity and promote confident communication with healthcare workers. Elimination of the fear of humiliation and rejection. To enhance their active participation in public health interventions without hesitancy.
	Welcoming Environment	Promoting LGBTQIA+ inclusivity in healthcare facilities, public places and virtual health centers by Displaying LGBTQIA+ symbols, diverse gender images, same gender images and LGBTQIA+ families. Designing a medical curriculum with the inclusion of LGBTQIA+-specific competencies. Distribution of brochures, magazines, posters and other reading materials from LGBTQIA+ organizations. Organizing special events that happen during pride month (June) to promote LGBTQ inclusivity and discuss their issues.	Creation of a gender-inclusive environment to enhance the interaction between the LGBTQIA+ population and the general public. Improved mental health of LGBTQIA+ individuals by making them feel more comfortable and safe among people. Enhanced privacy and individuality.
	Recruitment and retainment	Recruiting and retaining clinicians who hold proficiency in LGBTQIA+ healthcare or those who are highly interested in working towards their health benefits and inclusivity.	Enhanced education and mentorship programs that aim towards creating a safe and supportive environment for LGBTQIA+. Implementation of vaccine coverage strategies that include LGBTQIA+ communities in all healthcare services and newer health technology accessibility.
Community level	Partnership with LGBTQIA+ organizations	To increase awareness by building partnerships with local LGBTQIA+ organizations. To conduct events in collaboration with them on nationally recognized LGBTQIA+ specific days like national coming out day, which is celebrated on 11 October, transgender day of remembrance on 20 November, LGBT health week in March and pride month in June, about the health issues and prevailing concerns over LGBTQIA+ in inclusivity and equity. To analyze the requirements of this community through focus groups, cross-sectional survey and communication with the chief LGBTQIA+ heads, stakeholders and policymakers.	Needs assessment of the individuals of this community and structuring plans to address the same with legal and ethical regulations. Promote an inclusive healthcare environment that will encourage the sexual and gender minorities to actively participate in health promotion schemes like vaccination drives without the fear of discrimination and stigma, thus promoting mass vaccination and strengthening the immunity of the LGBTQIA+ population in the community.

**Table 5:** Recommendations for “mHealth” app development and implementation.

Process of Developing mHealth App	Explanation
Efficient mobile application development team	A multidisciplinary team comprising software developers, LGBTQIA+ healthcare professionals, mHealth experts and individuals of the LGBTQIA+ community should be engaged in the app development process. To prioritize the needs of LGBTQIA+ individuals.
Effective designing of the mobile application	To develop a user-centered design. Ensuring the effective functionality and usability of the app. Maximizing the perceived benefits of using the application. Minimizing the perceived barriers and burdens of app usage. Economical and multi-linguistic.
Testing of the prototype application	The prototype app developed should be tested with participants representing the LGBTQIA+ community. A focus group discussion to identify their expectations, needs and preferences. The feedback received should be meticulously analyzed and incorporated into the application.
Components and features of the mobile application	Online registration portals for scheduling vaccination slots exclusively for the LGBTQIA+ population. The availability of vaccines customized as per user demographics. Dashboard providing updates and information regarding the users’ vaccination status. Reminders and notifications for scheduled vaccination slots or vaccine overdue. Chatbot feature for assisting LGBTQIA+ individuals in the clarification of queries and reporting adverse reactions. Factual and general information about coronavirus, the COVID-19 pandemic and global vaccination rates from reliable sources.
Ethical Considerations	Ensuring autonomy, beneficence, non-maleficence and justice. No breach in data privacy. Transparent data handling. Equality in access. Right-to-exit service.

**Table 6:** The major barriers to promoting the inclusivity and implementation of digital health technologies.

Intervention	Social and Cultural Barriers	Structural Barriers
Promoting Inclusivity	Lack of acceptance Lack of awareness Discrimination Social stigma Existing prejudices	Lack of funding support from organizations Leadership Non-supportive administration Recruitment of trained staff
Digital health interventions	Lack of trust Lack of awareness Conventional mindset Language issues Fear of data piracy Inaccessibility to technology	Lack of funding supposed to design, develop and deliver digital health technologies Administrative and governing support Approval of stakeholders and policymakers Ethical and legal considerations

## Data Availability

The data that support this study are available upon request from the corresponding author.
